# AMS 4.0: consensus prediction of post-translational modifications in protein sequences

**DOI:** 10.1007/s00726-012-1290-2

**Published:** 2012-05-04

**Authors:** Dariusz Plewczynski, Subhadip Basu, Indrajit Saha

**Affiliations:** 1 Interdisciplinary Centre for Mathematical and Computational Modelling, University of Warsaw, 5a Street, 02-106 Warsaw, Poland; 2 Department of Computer Science and Engineering, Jadavpur University, Kolkata, 700032 India

**Keywords:** Post-translational modifications, AMS-4, High quality indices, MLP, Consensus

## Abstract

We present here the 2011 update of the AutoMotif Service (AMS 4.0) that predicts the wide selection of 88 different types of the single amino acid post-translational modifications (PTM) in protein sequences. The selection of experimentally confirmed modifications is acquired from the latest UniProt and Phospho.ELM databases for training. The sequence vicinity of each modified residue is represented using amino acids physico-chemical features encoded using high quality indices (HQI) obtaining by automatic clustering of known indices extracted from AAindex database. For each type of the numerical representation, the method builds the ensemble of Multi-Layer Perceptron (MLP) pattern classifiers, each optimising different objectives during the training (for example the recall, precision or area under the ROC curve (AUC)). The consensus is built using brainstorming technology, which combines multi-objective instances of machine learning algorithm, and the data fusion of different training objects representations, in order to boost the overall prediction accuracy of conserved short sequence motifs. The performance of AMS 4.0 is compared with the accuracy of previous versions, which were constructed using single machine learning methods (artificial neural networks, support vector machine). Our software improves the average AUC score of the earlier version by close to 7 % as calculated on the test datasets of all 88 PTM types. Moreover, for the selected most-difficult sequence motifs types it is able to improve the prediction performance by almost 32 %, when compared with previously used single machine learning methods. Summarising, the brainstorming consensus meta-learning methodology on the average boosts the AUC score up to around 89 %, averaged over all 88 PTM types. Detailed results for single machine learning methods and the consensus methodology are also provided, together with the comparison to previously published methods and state-of-the-art software tools. The source code and precompiled binaries of brainstorming tool are available at http://code.google.com/p/automotifserver/ under Apache 2.0 licensing.

**Electronic supplementary material:**

The online version of this article (doi:10.1007/s00726-012-1290-2) contains supplementary material, which is available to authorized users.

## Background

Post-translational modification (PTM) is a chemical modification of a protein after its translation. During protein synthesis, a protein is built using basic blocks of twenty different amino acids. Then the process of modification is taking place by attaching to them other biochemical functional groups such as acetate, phosphate, various lipids and carbohydrates, by changing the chemical nature of an amino acid, or by making structural changes, like the formation of disulfide bridges. In the advent of massive next generation sequencing experiments, the availability of whole proteomes requires accurate computational techniques for investigation of protein modification sites in the high-throughput scale. To address these needs we present here the recent update of the AMS tool for identification of post-translational modification sites in proteins using only sequence information. The method is based on the consensus between efficiently designed artificial neural networks, trained on proteins from the current version of Swiss-Prot database (Bairoch and Apweiler 1999) and Phospho.ELM dataset (Diella et al. 2004, 2008). The earlier version of the PTM prediction software was released as AMS 3.0 web server (Basu and Plewczynski 2010), and attracted large interest among the scientific community, we observed large internet traffic on our web site http://code.google.com/p/automotifserver/. The popularity of AMS 3.0 software has prompted us to release an upgraded version of the software, powered by the high quality indices, physico-chemical features and the consensus meta-learning algorithm.

The automatic prediction of PTM sites is an important area of interest for the bioinformatics research community. The currently available PTM prediction tools can be mostly categorised on the basis of their respective classification methodologies and the scope of prediction. In addition some researchers have developed consensus based approaches, that combine several signature recognition methods to scan a given query protein sequence against observed protein signatures. PROSITE (Sigrist et al. 2002) and Sulfinator (Monigatti et al. 2002) are typical examples in this category.

The other popular techniques mostly involve artificial neural network, support vector machine, and other machine learning approaches to PTM site prediction. These include NetPhos (Blom et al. 1999) and NetPhosK (Blom et al. 2004; Hjerrild et al. 2004), NetOGlyc (Julenius et al. 2005), NetNGlyc, DictyOGlyc (Gupta et al. 1999), YinOYang (Gupta and Brunak 2002), PredPhospho (Kim et al. 2004), Scansite (Yaffe et al. 2001), GPS (Xue et al., 2005 2006), PHOSITE (Koenig and Grabe 2004), KinasePhos 2.0 (Wong et al. 2007), etc. Our previously developed web server AutoMotifServer (AMS) (Plewczynski et al. 2005) for prediction of post-translational modification sites in protein sequences also uses SVM classifier with both linear and polynomial kernels. The software was available freely only as the web server at http://ams2.bioinfo.pl/. The currently available version of our AutoMotif Server (AMS-3) (Basu and Plewczynski 2010) software predicts large set of PTM types using MLP based predictors. More detailed work was done for acetylation prediction by (Xu et al. 2010; Gnad et al. 2010) and (Li et al. 2009), who developed lysine acetylation prediction tools using the SVM classifier. The recent work of Wan et al. (2008) designed an efficient meta-predictor that organise and process predictions from individual source prediction algorithms. They compiled and evaluated their technique on four unbiased phosphorylation site datasets, namely the four major protein kinase families: CDK, CK2, PKA and PKC.

**Fig. 1:**
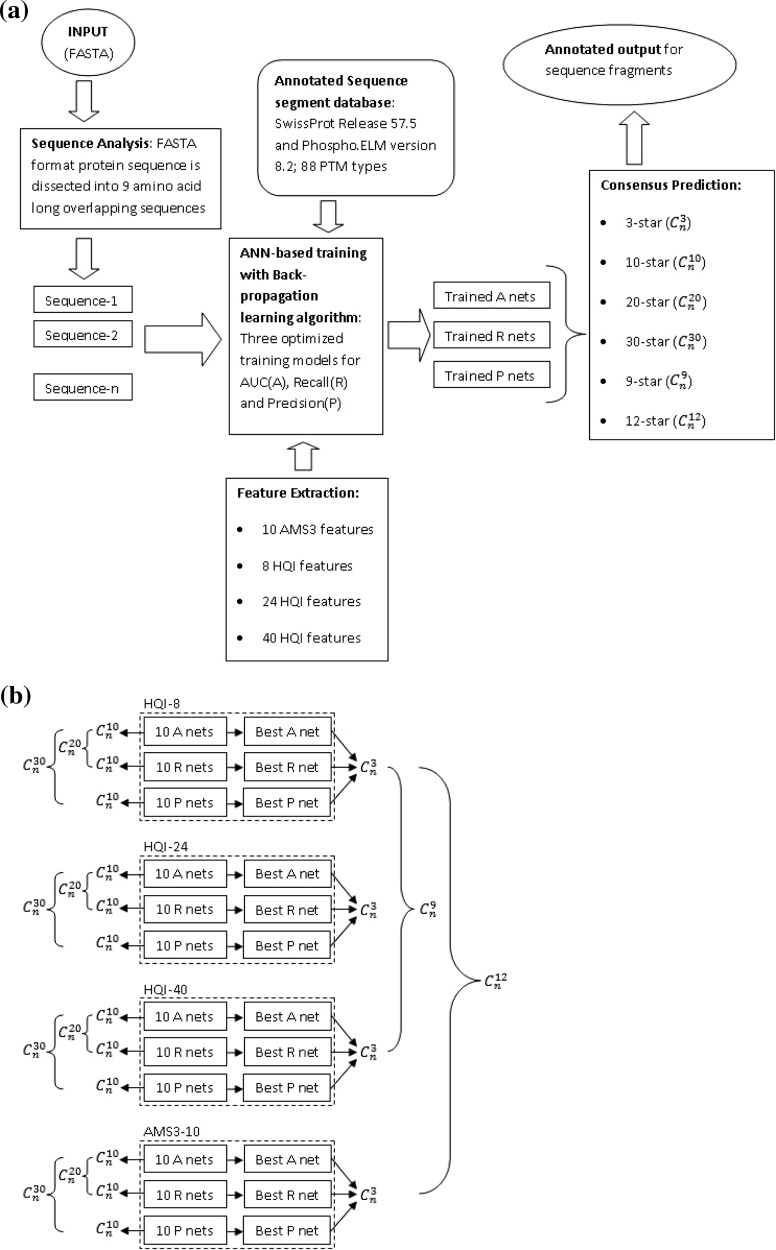
**a** The schematic block diagram of the consensus based prediction server for Post-Translational Modification sites in Protein sequences. **b** a detailed description of the consensus algorithm is shown. The input FASTA format protein sequence is dissected into 9 amino acid long overlapping sequences. Annotated sequence segment databases for 88 PTM types are collected from the recent versions of Swiss-Prot and Phiospho.ELM databases. Features are extracted from AAIndex database release 9.0. Three sets of MLP based classifiers are then trained to generate AUC, Recall and Precision optimised prediction results. Six different consensus schemes are then designed to integrates the set of differently optimised predictors into the single meta-learning predictor, and is able to boost the prediction performance in comparison with the single classification methods

Despite almost a decade of research on computational solutions for this problem, there is still a room for improvement of the precision of in silico methods. The complex nature of functional sequence motifs influences strongly the quality of classification, therefore impacting negatively the prediction accuracy, to be more useful in high-throughput context of systems biology studies. In this paper, we present the consensus approach that is based on fast machine learning method, namely, Multi-Layer Perceptron (MLP) artificial neural network (Rumelhart et al. 1985), along with diverse sets of most informative amino acids features selected by high quality indices clustering. More specifically, the current work focuses on: (1) clustering of amino acid indices features in three sets of high quality indices (HQIs), comprising of 8, 24 and 40 different features respectively. These three sets of feature vectors are subsequently referred as HQI-8, HQI-24 and HQI-40 respectively in the rest of the manuscript, (2) estimate the average and the best performances of the recall, precision and AUC optimised MLP predictors on test datasets of 88 different PTM types, separately using HQI-8, HQI-24 and HQI-40 feature vectors, along with the previously used 10 AMS-3 features (referred as AMS3-10 in rest of the manuscript) described in (Basu and Plewczynski 2010), (3) for each of those amino acids representations we employ six different consensus strategies among the saved networks for the best recall, precision and AUC optimised predictors, using the features described as AMS3-10, HQI-8, HQI-24 and HQI-40. The schematic block diagram of the developed consensus based prediction technique is shown in Fig. 1a, b. The accuracy of new method is significantly larger, when comparing with the previous versions of AMS prediction tool (Basu and Plewczynski 2010). The brainstorming consensus between efficiently designed MLP pattern classifiers and diverse physico-chemical representations is capable of classifying highly complex and nonlinear biological sequence motifs, where non-trivial and weak correlations between amino acid positions and types are important. The proposed meta-learning approach hierarchically improves the quality of predictions by combining results of several, differently optimised sub-methods.

## Methods

We used as the training dataset proteins extracted from the Swiss-Prot Release 57.5 (consisting of 470,369 entries), and Phospho.ELM dataset version 8.2 downloaded from http://phospho.elm.eu.org/dataset.html web site. Phospho.ELM version 8.2 contains 4,687 substrate proteins covering 2,217 tyrosine, 14,518 serine and 2,914 Threonine instances.

In our approach, the query protein sequence is dissected into overlapping short sequence segments. Each segment is represented using a vector of numerical values, where each amino acid is described using its physico-chemical characteristics. The database of AAindex (http://www.genome.jp/aaindex/) has been used to analyse by recently developed consensus fuzzy clustering technique for generating the subsets of HQIs (Saha et al. 2011). AAindex is a database of numerical indices representing various physico-chemical and biochemical properties of amino acids and pairs of amino acids. In 1988, Nakai et al. (Nakai et al. 1988) came up with 222 amino acid indices from published literature and investigated the relationships among them using hierarchical clustering analysis. Subsequently, Tomii and Kanehisa (Tomii and Kanehisa 1996) enriched the AAindex database with 42 amino acid mutation matrices and released as the AAindex2. Recently, 47 amino acid contact potential matrices have been reported as AAindex3. The database is continuously updated by Kawashima et al. (Kawashima et al. 1999, 2008; Kawashima and Kanehisa 2000). Currently, 544 amino acid indices are released in AAindex1 database.

However, the selection of the minimal/optimal set of amino acid indices for different bioinformatics applications is a difficult task and often involves adhoc/sub-optimal choices. It is therefore necessary to group similar indices in clusters and label representative cluster-indices. Moreover, the clustering of Amino acid indices done previously by Tomii et al. (Tomii and Kanehisa 1996; Kawashima et al. 2008) categorised 402 indices into six groups by using hierarchical clustering technique. Those clusters/groups represent Alpha and turn propensities, Beta propensity, Composition, Hydrophobicity, Physico-chemical properties and other properties. However, 142 amino acid indices of current database have not been clustered. These facts motivated us to analyse the current AAindex database using consensus fuzzy clustering, which we believe better describe the complex nature of chemical and physical similarity between amino acids. The consensus fuzzy clustering technique has been developed by using the majority voting of all recently proposed fuzzy clustering techniques (Bezdek 1981; Krishnapuram et al. 1999; Maulik and Bandyopadhyay 2003; Maulik and Saha 2009, 2010; Mauliket al 2010). After clustering of enhanced AAindex database, we have found three (3) new clusters, overall eight (8) clusters, named as Electric properties, Hydrophobicity, Alpha and Turn propensities, Physico-chemical properties, Residue propensity, Composition, Beta propensity and Intrinsic propensities. The detail description of the clustering method, clustering software and supplementary material with clustering quality results are given at http://sysbio.icm.edu.pl/aaindex/AAindex/ (Saha et al. 2011).

**Fig. 2:**
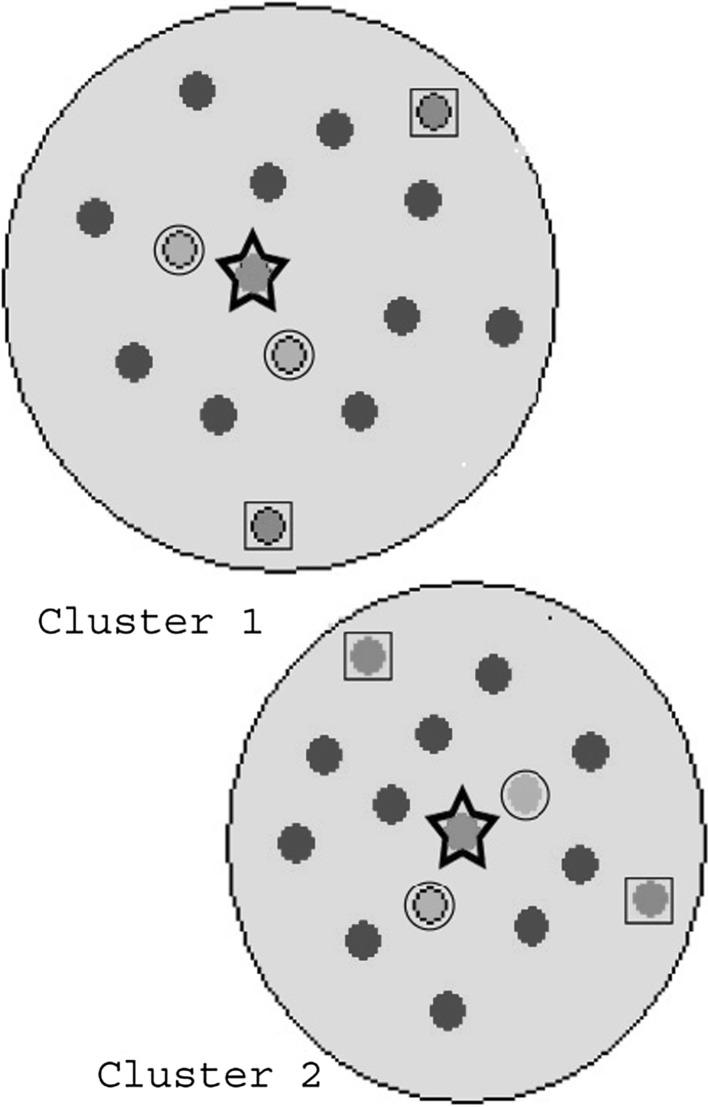
Illustrated the computational procedure of HQIs for two clusters, ‘star’ points are considered for HQI-2, ‘star + square’ points are considered for HQI-6, and ‘star + square + circle’ points are considered for HQI-10. In our case, number of clusters is 8, hence, we got HQI-8, HQI-24 and HQI-40

In order to provide the HQIs for the consensus fuzzy clustering results, three different approaches are used, which provide three different subsets of indices from the large AAindex database. For computing the high quality indices 8 (HQI-8), medoid (centre) of eight clusters is considered, which gives us indices called BLAM930101, BIOV880101, MAXF760101, TSAJ990101, NAKH920108, CEDJ970104, LIFS790101, MIYS990104. Similarly, for HQI-24 and HQI-40, three and five indices are considered from each cluster, respectively. For computing HQI-24, including the cluster medoid, other two farthest indices from the medoid are taken for each cluster. These two farthest indices are less significant for that cluster. However, they give more diversable properties of amino acid to that subset. Similarly for HQI-40, including the indices covered by the HQI-24 for all clusters, other two nearest indices of the medoid are considered from each cluster, that give strength to the property of medoids indices. All of these high quality indices HQI-8, HQI-24 and HQI-40 are separately mentioned in the supplementary (http://sysbio.icm.edu.pl/aaindex/AAindex/) with their amino acid values. The above procedure of computing HQIs is shown in Fig. 2 (Saha et al. 2011).

The identification of PTMs for each sequence segment of the query protein is done using the set of feed-forward artificial neural networks (ANN), which are trained with Back-Propagation (BP) learning algorithm (Rumelhart et al. 1986) to optimise the classification accuracy between the positive and the negative samples in the randomly chosen training subset of sequence segments. The optimisation procedure is tuned to produce three different ANNs, namely separately maximising the Recall (R), Precision (P), and the AUC (A) values for the training dataset chosen for each of the PTM type. For example, for PTM type Phospho_PKA, 861 positive data samples are generated. Each such data is represented as a 9 residues long sequence. Negative data samples are taken from fragments of sequences, where no known PTMs are observed. In order to generate the train and test samples for classification experiment, 577 samples (67 % of 861) are randomly selected as training patterns, and the rest 284 samples are considered as test set. In both train and test datasets the ratio between the positive and negative data samples are maintained as 1:5. Then we extract appropriate features (AMS3-10, HQI-8 etc.) for each of the data sequences. An MLP classifier with BP learning strategy is then trained for a fixed number of iterations over the training data samples, to finally predict the test patterns. The hidden neurons (in the only hidden layer) are varied from 2 to 20 in steps of 2. During the training phase, we optimise the network (i.e., adjust interconnection weights) to generate optimum Recall, Precision and AUC scores. The training is continued for a fixed number of iterations. To avoid over-fitting, network weights are saved as intermediate files at different stages of training. The network with best performance (among the set of intermediate networks generated at different iterations during training) over the test set is finally reported in this manuscript. In the training process, it may so happen that an intermediate network generated at a lower iteration may finally get selected as the best network in that specific training process. Please note that the test data was never used during the training and update of the network weights. All the experiments in the current work are run separately to train the A/R/P networks using the AM3-10, HQI-8, HQI-24 and HQI-40 feature sets. A detailed discussion involving the design issues of the MLP classifiers and A/R/P optimisation strategies are discussed in Basu and Plewczynski (2010).

To develop the consensus strategy for the current work, we assign the *n*-*star* quality result (*positive* prediction score) to any test sequence, where *n* is the number of optimised ANNs (trained networks) agreeing for the sequence fragment under consideration to be *positive* for a specific PTM type. For example, when a test sequence is classified as *positive* by all the trained neural networks under consideration, the strength of *positive* prediction is said to be of *n*-*star* quality. In contrast, if only one network predicts the test sequence to be *positive* the prediction quality is *1*-*star*. For test of unknown sequences, an end-user may tune the quality of prediction by choosing a specific value of *n.* Please note that, for any value of *n* = *m*; *m* ≥ 1, quality consensus of the order *(m* − *1)*-*star* are considered as *negative* predictions. Now we proceed with the aforementioned *n*-*star* quality consensus strategy with different sets of input networks. Here we have worked with six different sets of input network variations, by considering different sets of networks generated by AUC, Recall and Precision optimised trainings for each PTM type.

Agreement over classification decisions is achieved by (1) combining prediction decisions of different trained neural networks generated by varying the number of hidden neurons in each of the optimisation categories A, R and P. Since we varied the hidden neurons from 2 to 20, 10 trained networks are generated in each of the A, R and P optimisation experiments. Therefore, we implement a *10*-*star* consensus scheme for each of A, R and P optimisation schemes. (2) Combining prediction decisions of all neural networks obtained from A and R, thereby working on 20 trained networks in a *20*-*star* consensus scheme. (3) Combining prediction decisions of all neural networks obtained from A, R and P, i.e., a *30*-*star* consensus scheme. (4) Combining prediction decisions of three best performing neural networks obtained from A, R and P, a *3*-*star* consensus. (5) In another consensus scheme, we combine networks across different feature descriptors. 3 best performing networks from A, R and P optimisations are considered for each of the 3 feature descriptors, HQI-8, HQI-24 and HQI-40. This gives 9 networks for a *9*-*star* consensus scheme. (6) In another variation of the previous consensus approach, we combine 12 best performing networks obtained using HQI-8, HQI-24, HQI-40 and AMS3-10 feature descriptors.

The consensus procedures designed in our work address specific requirements from the biologists, generating high recall/precision values for any given query sequence, using respective recall/precision optimised network setups. In addition, the network setup for optimum AUC area gives a balanced prediction for query sequence, resulting in moderately high (optimum) recall/precision values. The classification results are generated along with a probabilistic confidence measure for such decision. The schematic block diagram of the designed consensus based PTM prediction scheme is shown in Fig. 1a, b. In the following section we describe the detailed theory and notations involved in implementation of the abovementioned consensus algorithm.

## Consensus

In general, we define a *n*-*star* quality consensus scheme as *C*_*n*_^N^, where N is the number of neural networks participating in the specific consensus strategy, and *n*(1 ≤ *n* ≤ N) is the quality of prediction. More specifically, 1-*star* prediction says that any one of possible N networks predicts the test sequence to be *positive* for the PTM type under consideration, and N-*star* represents that *all* networks agreed to the decision. Along this principle, we define the *10*-*star* quality consensus prediction $C_{n}^{10}$ as the consensus over 10 variations of hidden neurons (hidden neuron counts 2–20 in steps of 2) for AUC based optimisation. Similarly, we define $C_{n}^{20}$ and $C_{n}^{30}$ that combine 20 network predictions from A and R, and 30 network predictions from A, R and P respectively. Subsequently, $C_{n}^{3}$ is defined as the consensus among three best A, R, P networks, as described in (4) above (in Methods section). $C_{n}^{9}$ and $C_{n}^{12}$ are defined as the consensus over the best networks across different feature sets, as discussed in (5) and (6) respectively. In the following we first discuss the $C_{n}^{10}$ consensus algorithm and then describe the other variations.

Let $n_{k}^{\text{A}} ,n_{k}^{\text{R}} ,n_{k}^{\text{P}}$ be the MLP networks with *k* neurons in the hidden layer, designed to generate optimum AUC score (A), Recall (R) and Precision (P) scores respectively over the test dataset. Let $p_{k}^{\text{A}} ,p_{k}^{\text{R}} ,p_{k}^{\text{P}}$ be the prediction results corresponding to the networks $n_{k}^{\text{A}} ,n_{k}^{\text{R}} ,n_{k}^{\text{P}}$ for any unknown test pattern, where:

$$ p_{k}^{A} = \left\{ {_{{0;\quad {\text{otherwise}}}}^{{1;\quad {\text{test}}\,{\text{pattern}}\,{\text{is}}\,{\text{classified}}\,{\text{as}}\,{\text{positive}}\,{\text{by}}\,n_{k}^{A} }} } \right. $$

Similarly $p_{k}^{\text{R}} ,p_{k}^{\text{P}}$ also generate binary prediction decisions based on the classification confidence of the corresponding MLP classifiers $n_{k}^{\text{R}}$ and $n_{k}^{\text{P}}$ respectively. Now the general *n*-*star* consensus is designed as $C_{n}^{\text{N}}$, where *n* = minimum number of networks advocating for a test fragment to be positive. The sum of prediction scores is defined as $S_{p}^{\text{N}}$. For example, in case of $C_{n}^{ 1 0}$ if, $S_{p}^{10} = \mathop \sum \limits_{k} p_{k}^{\text{A}} ; k = 2\, to\, 20\, in\, steps\, of\, 2$, a test pattern is said to be predicted with *n*-*star* quality if $n \le S_{p}^{10}$. Similarly, for $C_{n}^{ 2 0}$, we estimate $S_{p}^{20} = \sum\nolimits_{k} {p_{k}^{\text{A}} } + \sum\nolimits_{k} {p_{k}^{\text{R}} }$ and for $C_{n}^{ 3 0}$, $S_{p}^{30} = \sum\nolimits_{k} {p_{k}^{\text{A}} } + \sum\nolimits_{k} {p_{k}^{\text{R}} } + \sum\nolimits_{k} {p_{k}^{\text{P}} }$, where $k = 2 \,to\, 20\, in\, steps\, of\, 2$ in all cases.

For $C_{n}^{ 3}$ we first define a function ${\text{M}}ax\_{\text{AUC}}\_over\_{\text{T}}estdata$ (MAT) to select the best performing network in any given optimisation category. The performance is evaluated in terms of maximum AUC score over the test dataset, as already discussed above. Therefore, we first compute $n_{\text{MAT}}^{\text{A}} = {\text{MAT}}\left( {n_{k}^{\text{A}} } \right);k = 2 \, to \, 20 \, in \, steps \,of \, 2$. Similarly, we compute $n_{\text{MAT}}^{\text{R}} = {\text{MAT}}\left( {n_{k}^{\text{R}} } \right) \, and \, n_{\text{MAT}}^{\text{P}} = {\text{MAT}}\left( {n_{k}^{\text{P}} } \right)$. The corresponding prediction scores are for the three selected networks are defined as $p_{\text{MAT}}^{\text{A}} ,p_{\text{MAT}}^{\text{R}}$ and $p_{\text{MAT}}^{\text{P}}$ respectively and the sum of prediction scores as, $S_{p}^{3} = p_{\text{MAT}}^{\text{A}} + p_{\text{MAT}}^{\text{R}} + p_{\text{MAT}}^{\text{P}}$.

In the case of $C_{n}^{9}$ we use the MAT function separately for the three different feature sets under consideration for the current work, viz*.*, HQI-8, HQI-24 and HQI-40. Therefore we define the function ${\text{MAT}} - {\text{HQI}} - 8$ to generate three best performing nets as $n_{{{\text{MAT}} - {\text{HQI}} - 8}}^{\text{A}} = {\text{MAT}} - {\text{HQI}} - 8\left( {n_{k}^{\text{A}} } \right); k = 2\, to\, 20\, in\, steps\, of\, 2$, and likewise $n_{{{\text{MAT}} - {\text{HQI}} - 8}}^{\text{R}}$ and $n_{{{\text{MAT}} - {\text{HQI}} - 8}}^{\text{P}}$. In the same way three best networks are generated by each of the functions ${\text{MAT}} - {\text{HQI}} - 24$ and ${\text{MAT}} - {\text{HQI}} - 40$. The sum of the corresponding prediction scores is then defined as:

$$ \begin{gathered} S_{p}^{9} = p_{{{\text{MAT}} - {\text{HQI}} - 8}}^{\text{A}} + p_{{{\text{MAT}} - {\text{HQI}} - 8}}^{\text{R}} + p_{{{\text{MAT}} - {\text{HQI}} - 8}}^{\text{P}} + p_{{{\text{MAT}} - {\text{HQI}} - 24}}^{\text{A}} + \hfill \\ p_{{{\text{MAT}} - {\text{HQI}} - 24}}^{\text{R}} + p_{{{\text{MAT}} - {\text{HQI}} - 24}}^{\text{P}} + p_{{{\text{MAT}} - {\text{HQI}} - 40}}^{\text{A}} + p_{{{\text{MAT}} - {\text{HQI}} - 40}}^{\text{R}} + \hfill \\ p_{{{\text{MAT}} - {\text{HQI}} - 40}}^{\text{P}} . \hfill \\ \end{gathered} $$

Similarly, for $C_{n}^{12}$ we use four different MAT functions separately for the four different feature sets, viz., ${\text{MAT}} - {\text{HQI}} - 8,\,{\text{MAT}} - {\text{HQI}} - 24,\,{\text{MAT}} - {\text{HQI}} - 40$ and ${\text{MAT}} - {\text{AMS}}3 - 10$. The sum of the corresponding prediction scores is then defined as:

$$ \begin{gathered} S_{p}^{12} = p_{{{\text{MAT}} - {\text{HQI}} - 8}}^{\text{A}} + p_{{{\text{MAT}} - {\text{HQI}} - 8}}^{\text{R}} + p_{{{\text{MAT}} - {\text{HQI}} - 8}}^{\text{P}} + p_{{{\text{MAT}} - {\text{HQI}} - 24}}^{\text{A}} + p_{{{\text{MAT}} - {\text{HQI}} - 24}}^{\text{R}} \hfill \\ + p_{{{\text{MAT}} - {\text{HQI}} - 24}}^{\text{P}} + p_{{{\text{MAT}} - {\text{HQI}} - 40}}^{\text{A}} + p_{{{\text{MAT}} - {\text{HQI}} - 40}}^{\text{R}} + p_{{{\text{MAT}} - {\text{HQI}} - 40}}^{\text{P}} + p_{{{\text{MAT}} - {\text{AMS}}3 - 10}}^{\text{A}} \hfill \\ + p_{{{\text{MAT}} - {\text{AMS}}3 - 10}}^{\text{R}} + p_{{{\text{MAT}} - {\text{AMS}}3 - 10}}^{\text{P}} . \hfill \\ \end{gathered} $$

As discussed before, *n*-*star* quality result is obtained for any specific PTM type between the ANN networks in any of the six ways, viz., $C_{n}^{10} ,C_{n}^{20} ,C_{n}^{30} ,C_{n}^{3} ,C_{n}^{9} \,{\text{or}}\,C_{n}^{12}$. We assign the statistical significance based on “how many ANNs agree that selected fragment is predicted as *Positive* for a PTM type”. Implementation and performances of these consensus approaches are discussed in details in the following section.

## Results and discussion

In the current work we have implemented multiple consensus schemes to improve the recognition accuracy of the existing A/R/P optimised single network accuracies. Detail experiment with all the positive samples for each of 88 PTM types is conducted to validate the findings. The experiment is conducted with the optimised AUC, Recall and Precision networks over 10 different hidden neuron variations for each PTM type during the training process. System and methods related to these optimum single networks are reported in one of our recent works (Basu and Plewczynski 2010). AUC, Recall and Precision performances corresponding to the training and test datasets of 88 different PTM types is given in the supplementary excel sheet. The objective of the current work is to design a consensus based meta-prediction scheme over such trained networks. To compare the current results with the single network performances only the AUC values are considered. Detailed experimental results for *n*-*star* quality predictions for $C_{n}^{10} ,C_{n}^{20} ,C_{n}^{30} ,C_{n}^{3} ,C_{n}^{9}$ and $C_{n}^{12}$ consensus schemes are given in the supplementary excel sheet. Table 1 shows overall comparison of single network performances with the variations of *n*-*star* consensus results for 15 most promising PTM types, where significant performance gains are observed. It may also be observed from the experiments that the consensus strategy improves the prediction performances for almost all the PTM types, considered for the current work.

**Table 1:** Experimental results on 15 important PTM types are shown, where the developed consensus scheme is found to be significantly improving the corresponding AUC scores of the best single network based prediction strategies

PTM	Single network		AMS-4 Meta-Consensus	Gain over AMS-3 (%)	
	AMS-3				
	Average	Maximum	Maximum	Average	Maximum
Phosphothreonine_CDC2	0.685068	0.698365	0.910212	32.864475	30.33471
GRK_group	0.614195	0.693856	0.776483	26.422879	11.908379
CK1_group	0.4375	0.4375	0.541667	23.8096	23.8096
AMPK_group	0.769388	0.77551	0.94898	23.342189	22.368506
Abl	0.689333	0.693333	0.833333	20.889759	20.192317
Lyn	0.676389	0.680556	0.805556	19.096555	18.367335
Phosphoserine	0.734679	0.769004	0.865732	17.838131	12.578348
Tyrosine	0.81172	0.827492	0.954545	17.595353	15.353985
PLK1	0.729353	0.743781	0.854892	17.212379	14.938671
MAPK14	0.738125	0.74375	0.8625	16.850127	15.966387
GSK-3_group	0.747253	0.747253	0.870879	16.544062	16.544062
PDK-1	0.7375	0.8125	0.854167	15.819254	5.1282462
MAPKAPK2	0.647619	0.649471	0.743386	14.787553	14.46023
ATM	0.83347	0.842033	0.950549	14.047176	12.887381
Syk	0.685417	0.6875	0.770833	12.461903	12.121164

We have also compared the performance of the current experiment with the existing software tools, viz., GPS, KinasePhos, NetPhosK, PPSP, PredPhospho, Scansite and the Meta-predictor tool, along with our previously developed AMS-3 software. Four significant PTM types, CDK_group, CK2_group, PKA_group and PKC_group are considered for this benchmark comparison. The designed consensus strategy improves the recognition performance of the existing AMS-3 software in case of most PTM types under consideration. Details of this experiment are shown in Table 2. Apart from our AMS-3 tool, the PPSP, NetPhosK and Meta-predictor tools came in comparison with the developed AMS-4 software, with respect to the reported AUC scores. In fact, the performance of AMS-4 is less than NetPhosK and Meta-predictor scores in the case of the CK2_group. Furthermore, in the case of the PKA_group, the performances of AMS-4 and Meta-predictor are found to be at par. PPSP scores are also found to be close to the AMS-4 performances for the PKA_group and the CK2_group. However, for the PTM types, CDK_group and PKC_group, AMS-4 performance is found to be higher than the other tools under consideration. Overall, it may fairly be assessed that the performance of the new AMS-4 software is noteworthy and comparable with the existing software tools in this domain. In case of Lysine acytelation predictions, the current AMS-4 software also performs satisfactorily in comparison with some of the tools dedicated for the said prediction purpose. The average Recall/Sensitivity reported in (Xu et al. 2010) is in the range of 80 %. Similarly (Gnad et al. 2010) have used SVM to predict acetylated residues and reported Recall of 78 % on input data containing equal numbers of modified and non-modified residues. Acetylation prediction on lysine residues in (Li et al. 2009) has shown accuracies in the range 75–77 % using SVM pattern classifier. In the current work we predict *acetyllysine* PTM type with over 90 % Recall, Precision and AUC scores. Although the comparison is not performed on an identical test dataset, it may safely be concluded that the current consensus approach performs satisfactorily for acetylation predictions as well.

**Table 2:** Performance (AUC score) of the current AMS 4.0 experiment, for some of the key PTM types is compared with the existing *state*-*of*-*the*-*art* software tools

	CDK_group	CK2_group	PKA_group	PKC_group
GPS	*0.87*	*0.81*	*0.84*	*0.75*
KinasePhos	*0.87*	*0.75*	*0.82*	*0.74*
NetPhosK	*0.77*	***0.93***	*0.87*	*0.75*
PPSP	*0.87*	*0.87*	*0.88*	*0.79*
PredPhospho	*0.86*	*0.77*	*0.85*	*0.71*
Scansite	*0.75*	*0.77*	*0.76*	*0.63*
Meta-predictor	*0.89*	***0.93***	***0.89***	*0.82*
AMS 3.0	0.92	0.87	0.88	0.84
AMS 4.0	**0.95**	0.88	**0.89**	**0.86**

The highest performances are highlighted corresponding to each PTM type

The current experimental protocol improves the performance of our previously designed AMS 3.0 tool by more than 6 % on average (over all the 88 PTM types). Please note that, for many PTM types the prediction accuracy was already in the nineties, thereby having limited scope in increment of performance numbers. Keeping that in mind, an average performance increment of 6 % may be considered significant. The developed AMS 4.0 tool is a big step ahead of our previous AMS 3.0 tool. The key improvements are, (1) development of a wide variety of consensus strategies to combine the strength of multiple single networks (MLP based classifiers) to boost the prediction performance for a wide variety of PTM types, (2) clustering of amino acid physico-chemical features (http://sysbio.icm.edu.pl/aaindex/AAindex/), categorise them as three different indices sets, viz*.*, HQI-8, HQI-24 and HQI-40, and use them prudently for solving the problem under consideration, (3) development of a consensus among the heuristically chosen AMS 3.0 features, and the three sets of HQI features, and (4) development of a meta-consensus strategy by selecting the best approach for each PTM type.

In the current work, we first employ the consensus strategy over the existing classifiers, designed for the AMS 3.0 tool. The average AUC performances of $C_{n}^{10} ,C_{n}^{20} ,C_{n}^{30}$ and $C_{n}^{3}$ based consensus strategies are compared with the corresponding single network performances. More specifically, the AMS 4.0 consensus predictions for each PTM are compared with two different AMS 3.0 performance measures, viz*.*, (1) average AUC score over 10 different variations of hidden layer neurons for the MLPs, and, (2) maximum AUC score over the 10 variations. In the same way, we compare the AMS 4.0 performance (for $C_{n}^{10} ,C_{n}^{20} ,C_{n}^{30}$ and $C_{n}^{3}$) with the single network performances corresponding to the HQI-8, HQI-24 and HQI-40 feature sets. It may be observed from the detailed comparison table, given in the supplementary excel sheet, that the average of *average* AMS 3.0 AUC scores over 88 PTM types is around 83.45 %, while the average of *maximum* AUC scores is 84.20 %. Using consensus prediction over AMS 3.0 results, the average AUC score could be enhanced by around 2 %. The $C_{n}^{30}$ consensus strategy is found to be superior among the four contender consensus schemes. The average AUC score of 85.88 % is achieved in case of $C_{n}^{30}$ over AMS 3.0 results. The average $C_{n}^{30}$ AUC scores over HQI-8, HQI-24 and HQI 40 feature sets are reported as 84.05, 84.88 and 85.57 %, an improvement of around 2 % in corresponding single network performances. Although the average benefit of the use of HQI features over AMS 3.0, are not so apparent from the average consensus results, the choice of HQI features contributed in specific PTM types with significant gains. In addition, we have designed the $C_{n}^{9}$ and $C_{n}^{12}$ consensus strategies by combining classifiers from different feature combinations. The first scheme combines all the three HQI feature combinations and the later combines all the four, viz*.,* AMS3-10, HQI-8, HQI-24 and HQI-24. The average AUC score of 87.79 % is achieved for $C_{n}^{12}$ (best among the six consensus schemes and a gain of around 4 % over average AMS 3.0 performance). Finally, a meta-consensus strategy identifies the best scheme (among the possible six) for each of the PTM types, and the average AUC score of 88.79 % is achieved.

It may be worth mentioning in this context that the recognition performances reported in the original AMS 3.0 work (Basu and Plewczynski 2010) are not used in the current work for the comparison purpose. This is primarily because the sharp difference in the two experimental protocols. Current version of the dataset is very different from the earlier one and incorporates newer/additional variations of positive samples for most PTM types. In addition, the earlier dataset had many redundancies in positive samples (similar short sequences amino acid collected from different proteins), which are completely removed in the current dataset. Therefore, to compare the performance of AMS 4.0 we have recomputed the complete AMS 3.0 experiment to develop the new test-bed for performance evaluation.

We also compute performance gains for individual PTM types by comparing the meta-consensus AUC score with, (1) the corresponding average AMS 3.0 score for the PTM type, and (2) the maximum AMS 3.0 AUC score. From the supplementary sheets and from Table 1, it may be observed that up to 32 % performance gain (with respect to the average AMS 3.0 AUC score) could be achieved using the AMS 4.0 tool. More than 10 % average performance gains could be achieved for 21 PTM types. Overall, 6.94 % performance gain is observed for 88 PTM types. The average gains with respect to the maximum AUC scores of AMS 3.0 tool is estimated as 5.88 %. As for example, for the PTM type *Phosphothreonine_CDC2* an average performance gain of 32.86 % is observed. The corresponding gain with respect to the maximum AMS 3.0 AUC score is 30.33 %. The average AUC score is improved from 68.5 % to 91.02 % in this case. For PTM types GRK_group, CK1_group, AMPK_group and Abl over 20 % boost over average AUC score is observed. Key PTM types like Phospho_PKA, Phospho_PKC, Phospho_CDC2 and Phospho_auto have registered performance gains of 2.75, 2.79, 5.12 and 2.92 % over corresponding maximum AUC scores of AMS 3.0. In all these cases the AMS 3.0 performances were already in the range of 88-92 percent, thereby limiting the scope of high improvements. In case of some more PTM types like PKA_group, PKB_group, PKC_group, CDK_group and CK2_group the meta-consensus AUC scores could be enhanced up to 88.66, 93.38, 85.63, 95.01 and 88.23 %, respectively, with average gains of around 3 % over AMS 3.0. In general, for 88 different PTM types, performance gains could be achieved in almost all cases. However, for PTM types acetylglycine, Allysine, Cysteine_amide and Cysteine_methylsome, performance gain (over AMS 3.0) could not be achieved in the current work. Furthermore, in case of acetylserine and Pyrrolidone, the average performance could be improved marginally, but the maximum AUC score remains same as the corresponding AMS 3.0 scores. In future, we may need to explore these specific cases even further, by enriching the respective training and test databases and by selecting some additional features, to improve the results for these six PTM types.

## Conclusions

In the current work, we present the 2012 update of the AutoMotif Service (AMS) that predicts the wide selection of 88 different types of the single amino acid post-translational modifications (PTM) in protein sequences, using high quality indices (HQI) obtaining by automatic clustering of known indices extracted from AAindex database. In order to boost the overall prediction accuracy, a consensus is built using brainstorming technology that combines multi-objective instances of machine learning algorithm. Among different consensus strategies, the $C_{n}^{12}$ consensus scheme is found to give superior results in comparison to the *n*-*star* consensus schemes, thereby justifying the choice of HQI features. Our software improves the average AUC score of the earlier version by close to 7 % as calculated on the test datasets of all 88 PTM types. It may be worth mentioning in this context that the consensus strategy always retains the prediction quality of the single network based prediction schemes. The consensus meta-learning methodology on the average boosts the AUC score up to around 89 % over all PTM types. The overall boost is however moderate because of limited improvement potential of the highly optimised networks for many PTM types. In many cases, the AUC scores of such single networks are already in excess of 90 %. In a nutshell, significant performance improvement for most PTM types could be achieved in the AMS 4.0 software using the designed consensus strategy, without losing quality for the others, giving added value to the existing AMS 3.0 prediction software.

## Electronic supplementary material

Below is the link to the electronic supplementary material.

Supplementary material 1 (DOCX 144 kb)

Supplementary material 2 (XLSX 90 kb)
