# Supplementary material for: AMS 4.0: consensus prediction of post-translational modifications in protein sequences
Source: Amino Acids. 2012 May 4;43(2):573–82. doi: 10.1007/s00726-012-1290-2 (PMC3397139; doi:10.1007/s00726-012-1290-2)
Supplement: Supplementary file 1 — Supplementary material 1 (DOCX 144 kb) [file 726_2012_1290_MOESM1_ESM.docx]

# CONSENSUS ALGORITHM

To design the consensus strategy, we assign so-called *n-star* quality result to any test sequence, where *n* is the number of optimized ANNs (networks) agreeing for the sequence fragment under consideration to be modified by any preselected PTM type. Agreement over classification decision is achieved by (1) combining prediction decisions of different neural networks generated by varying the number of hidden neurons (k) in each of the optimization categories A, R and P, (2) combining prediction decisions of all neural networks obtained from A and R, (3) combining prediction decisions of all neural networks obtained from A, R and P, (4) combining prediction decisions of three best performing neural networks obtained from A, R and P. In another consensus approach we combine twelve best performing networks obtained (three best networks from each feature combination, as described in (4)) using HQI-8, HQI-24, HQI-40 and the 10 features used in AMS-3. The consensus procedures designed in our work address specific requirements from the biologists, generating high recall/precision values for any given query sequence, using respective recall/precision optimized network setups. Also, the network setup for optimum AUC area gives a balance prediction for query sequence, resulting in moderately high (balanced) recall/precision values. The classification results are generated along with a probabilistic confidence measure for such decision. The schematic block diagram of the designed consensus based PTM prediction scheme is shown in Figure 1(a-b).

In general, we define a $n-star$ quality consensus scheme as $C_{n}^{N}$, where $N$ is the number of neural networks participating in the specific consensus strategy, and $n (1\leq n\leq N)$ is the quality of prediction. More specifically, $1-star$ prediction says that any one (of possible N) networks agreed to the prediction decision, and $N-star$ represents that $all$ networks agreed to the decision. Along this principle, we define the *10-star* quality consensus prediction $C_{n}^{10}$as the consensus over 10 variations of hidden neurons (hidden neuron counts 2 to 20 in steps of 2) for AUC based optimization. Similarly, we define $C_{n}^{20}$and $C_{n}^{30}$that combine 20 network predictions from A and R, and 30 network predictions from A, R and P respectively. Subsequently, $C_{n}^{3}$ is defined as the consensus among three best A, R, P networks, as described in (4) above, and $C_{n}^{12}$ is defined as the consensus over the best networks across four different feature sets. In the following we first discuss the $C_{n}^{10}$consensus algorithm and then describe the other variations.

Let $n_{k}^{A},n_{k}^{R},n_{k}^{P}$ be the MLP networks with $k$ neurons in the hidden layer, designed to generate optimum AUC score (A), Recall (R) and Precision (P) scores respectively over the test dataset. Let $p_{k}^{A},p_{k}^{R},p_{k}^{P}$ be the prediction results corresponding to the networks $n_{k}^{A},n_{k}^{R},n_{k}^{P}$ for any unknown test pattern, where:

$$p_{k}^{A}=\left\{ \begin{matrix} 1; & test pattern is classified as positive by n_{k}^{A} \\ 0; & otherwise \end{matrix} \right.$$

Similarly $p_{k}^{R},p_{k}^{P}$also generate binary prediction decisions based on the classification confidence of the corresponding MLP classifiers $n_{k}^{R}$ and$n_{k}^{P}$ respectively. Now the general *n-star* consensus is designed as $C_{n}^{N}$, where $n=$ minimum number of networks advocating for a test fragment to be positive. The sum of prediction scores is defined as ${S^{N}}_{p}$. For example, in case of $C_{n}^{10}$ if, $S_{p}^{10}=\sum_{k} p_{k}^{A}; k=2 to 20 in steps of 2$, a test pattern is said to be predicted with $n-star$ quality if $n\leq S_{p}^{10}$. Similarly, for $C_{n}^{20}$, we estimate $S_{p}^{20}=\sum_{k} p_{k}^{A}+\sum_{k} p_{k}^{R}$ and for $C_{n}^{30}$, $S_{p}^{30}=\sum_{k} p_{k}^{A}+\sum_{k} p_{k}^{R}+\sum_{k} p_{k}^{P}$, where $k=2 to 20 in steps of 2$ in all cases.

For $C_{n}^{3}$we first define a function $Max\_AUC\_over\_Testdata$ (MAT) to select the best performing network in any given optimization category. The performance is evaluated in terms of maximum AUC score over the unbiased test dataset. Therefore, we first compute $n_{\mathrm{MAT}}^{A}=\mathrm{MAT}\left( n_{k}^{A} \right);k=2 to 20 in steps of 2$. Similarly, we compute $n_{\mathrm{MAT}}^{R}=\mathrm{MAT}\left( n_{k}^{R} \right) and n_{\mathrm{MAT}}^{P}=\mathrm{MAT}\left( n_{k}^{P} \right)$. The corresponding prediction scores are for the three selected networks are defined as $p_{\mathrm{MAT}}^{A},p_{\mathrm{MAT}}^{R}$ and $p_{\mathrm{MAT}}^{P}$ respectively and the sum of prediction scores as, $S_{p}^{3}=p_{\mathrm{MAT}}^{A}+p_{\mathrm{MAT}}^{R}+p_{\mathrm{MAT}}^{P}$.

In the case of $C_{n}^{12}$we use the $\mathrm{MAT}$ function separately for the four different feature sets under consideration for the current work, *viz.*, HQI-8, HQI-24, HQI-40 and AMS-3. Therefore we define the function $\mathrm{MAT}-\mathrm{HQI}-8$ to generate three best performing nets as $n_{\mathrm{MAT}-\mathrm{HQI}-8}^{A}=\mathrm{MAT}-\mathrm{HQI}-8\left( n_{k}^{A} \right); k=2 to 20 in steps of 2$, and likewise $n_{\mathrm{MAT}-\mathrm{HQI}-8}^{R}$ and $n_{\mathrm{MAT}-\mathrm{HQI}-8}^{P}$. In the same way three best networks are generated by each of the functions $\mathrm{MAT}-\mathrm{HQI}-24$, $\mathrm{MAT}-\mathrm{HQI}-40$ and $\mathrm{MAT}-\mathrm{AMS}-3$. The sum of the corresponding prediction scores is then defined as $S_{p}^{12}=p_{\mathrm{MAT}-\mathrm{HQI}-8}^{A}+p_{\mathrm{MAT}-\mathrm{HQI}-8}^{R}+p_{\mathrm{MAT}-\mathrm{HQI}-8}^{P}+p_{\mathrm{MAT}-\mathrm{HQI}-24}^{A}+p_{\mathrm{MAT}-\mathrm{HQI}-24}^{R}+p_{\mathrm{MAT}-\mathrm{HQI}-24}^{P}{+p}_{\mathrm{MAT}-\mathrm{HQI}-40}^{A}+p_{\mathrm{MAT}-\mathrm{HQI}-40}^{R}+p_{\mathrm{MAT}-\mathrm{HQI}-40}^{P}+p_{\mathrm{MAT}-\mathrm{AMS}-3}^{A}+p_{\mathrm{MAT}-\mathrm{AMS}-3}^{R}+p_{\mathrm{MAT}-\mathrm{AMS}-3}^{P}$.

As discussed before, *n-star* quality result is obtained for any specific PTM type between the ANN networks in any of the five ways. We assign the statistical significance based on "how many ANNs agree that selected fragment is predicted as *Positive* for a PTM type". The algorithm for n-star consensus scheme is discussed below:

- Consider any sequence fragment and select the PTM type.
- Accumulate the prediction decisions generated by the AUC, Recall and Precision optimized MLP networks corresponding to different hidden neuron variations.
- For any given consensus strategy ($C_{n}^{10}$,$C_{n}^{20}$,$C_{n}^{30}$,$C_{n}^{3}$ or $C_{n}^{12}$) count how many ANNs agree that selected fragment is predicted as *Positive* for a PTM type.
- Accept only those predictions that are confirmed by all networks.
- Rank the remaining predictions as *n-star* quality consensus, where *n* signifies the consensus count.

Let's assume that any selected fragment is predicted as PKA phosphorylated (say) by ANNs from all three networks A, R and P, then the consensus approach confirms that this fragment is phosphorylated by PKA and this fragment will be *3-star* quality.

Implementation and performances of these consensus approaches are discussed in details in the following section.

# IMPLEMENTATION

In the current work we have implemented multiple consensus schemes to improve the recognition accuracy of the existing A/R/P optimized single network accuracies. Detail experiment with all the positive samples for each of 88 PTM types is conducted to validate the findings. The experiment is conducted with the optimized AUC, Recall and Precision networks over 10 different hidden neuron variations for each PTM type during the training process. System and methods related to these optimum single networks are reported in one of our recent works (Basu, et al., 2010). AUC, Recall and Precision performances corresponding to the training and test datasets of 88 different PTM types is given in the supplementary excel sheet. The objective of the current work is to design a consensus based meta-prediction scheme over such trained networks. To compare the current results with the single network performances only the AUC values are considered. Detailed experimental results for *n-star* quality predictions for $C_{n}^{10}$,$C_{n}^{20}$,$C_{n}^{30}$,$C_{n}^{3}$, $C_{n}^{9}$ and $C_{n}^{12}$ consensus schemes are given in the supplementary excel sheet. Table 1 shows overall comparison of single network performances with the variations of *n-star* consensus results for all PTM types, with performance gains against AMS-3 tool. It may also be observed form the experiments that the consensus strategy improves the prediction performances for almost all the PTM types, considered for the current work.

We have also compared the performance of the current experiment with the existing software tools, viz., GPS, KinasePhos, NetPhosK, PPSP, PredPhospho, Scansite and the Meta-predictor tool, along with our previously developed AMS-3 software. Four significant PTM types, CDK_group, CK2_group, PKA_group and PKC_group are considered for this benchmark comparison. The designed consensus strategy improves the recognition performance of the existing AMS-3 software in case of most PTM types under consideration. Details of this experiment are shown in Table 2. Apart from our AMS-3 tool, the PPSP, NetPhosK and Meta-predictor tools came in comparison with the developed AMS-4 software, with respect to the reported AUC scores. In fact, the performance of AMS-4 is less than NetPhosK and Meta-predictor scores in case of CK2_group. Also in case of PKA_group the performances of AMS-4 and Meta-predictor are found to be at par. PPSP scores are also found to be close to the AMS-4 performances for PKA_group and CK2_group. However, for the PTM types CDK_group and PKC_group, AMS-4 performance is found to be higher than the other tools under consideration. Overall, it may fairly be assessed that the performance of the new AMS-4 software is noteworthy and comparable with the existing software tools in this domain. In case of Lysine acytelation predictions, the current AMS-4 software also performs satisfactorily in comparison with some of the tools dedicated for the said prediction purpose. The average Recall/Sensitivity reported in (Xu, et al., 2010) is in the range of 80%. Similarly (Gnad, et al., 2010) has used SVM to predict acetylated residues and reported Recall of 78% on input data containing equal numbers of modified and non-modified residues. Acetylation prediction on lysine residues in (Li, et al., 2009) has shown accuracies in the range of 75-77% using SVM pattern classifier. In the current work we predict *acetyllysine* PTM type with over 90% Recall, Precision and AUC scores. Although the comparison is not performed on an identical test dataset, it may safely be concluded that the current consensus approach performs satisfactorily for acetylation predictions as well.

# DISCUSSION

The current experimental protocol improves the performance of our previously designed AMS 3.0 tool by more than 6% on average (over all the 88 PTM types). Please note that, for many PTM types the prediction accuracy was already in the nineties, thereby having limited scope in increment of performance numbers. Keeping that in mind, an average performance increment of 6% may be considered significant. The developed AMS 4.0 tool is a big step ahead of our previous AMS 3.0 tool. The key improvements are, 1) development of a wide variety of consensus strategies to combine the strength of multiple single networks (MLP based classifiers) to boost the prediction performance for a wide variety of PTM types, 2) clustering of amino acid physico-chemical features (<http://sysbio.icm.edu.pl/aaindex/AAindex/>), categorize them as three different indices sets, *viz.*, HQI-8, HQI-24 and HQI-40, and use them prudently for solving the problem under consideration, 3) development of a consensus among the heuristically chosen AMS 3.0 features, and the three sets of HQI features, and 4) development of a meta-consensus strategy by selecting the best approach for each PTM type.

In the current work, we first employ the consensus strategy over the existing classifiers, designed for the AMS 3.0 tool. The average AUC performances of $C_{n}^{10}$,$C_{n}^{20}$,$C_{n}^{30}$and $C_{n}^{3}$ based consensus strategies are compared with the corresponding single network performances. More specifically, the AMS 4.0 consensus predictions for each PTM are compared with two different AMS 3.0 performance measures, *viz.*, 1) average AUC score over 10 different variations of hidden layer neurons for the MLPs, and, 2) maximum AUC score over the 10 variations. In the same way, we compare the AMS 4.0 performance (for $C_{n}^{10}$,$C_{n}^{20}$,$C_{n}^{30}$and $C_{n}^{3}$) with the single network performances corresponding to the HQI-8, HQI-24 and HQI-40 feature sets. It may be observed from the detailed comparison table, given in the supplementary excel sheet, that the average of *average* AMS 3.0 AUC scores over 88 PTM types is around 83.45%, while the average of *maximum* AUC scores is 84.20%. Using consensus prediction over AMS 3.0 results, the average AUC score could be enhanced by around 2%. The $C_{n}^{30}$consensus strategy is found to be superior among the four contender consensus schemes. The average AUC score of 85.88% is achieved in case of $C_{n}^{30}$ over AMS 3.0 results. The average $C_{n}^{30}$ AUC scores over HQI-8, HQI-24 and HQI 40 feature sets are reported as 84.05%, 84.88% and 85.57%, an improvement of around 2% in corresponding single network performances. Although the average benefit of the use of HQI features over AMS 3.0, are not so apparent from the average consensus results, the choice of HQI features contributed in specific PTM types with significant gains. In addition, we have designed the $C_{n}^{9}$ and $C_{n}^{12}$ consensus strategis by combining classifiers from different feature combinations. The first scheme combines all the three HQI feature combinations and the later combines all the four, *viz.,* AMS3-10, HQI-8, HQI-24 and HQI-24. The average AUC score of 87.79% is achieved for $C_{n}^{12}$ (best among the six consensus schemes and a gain of around 4% over average AMS 3.0 performance). Finally, a meta-consensus strategy identifies the best scheme (among the possible six) for each of the PTM types, and the average AUC score of 88.79% is achieved.


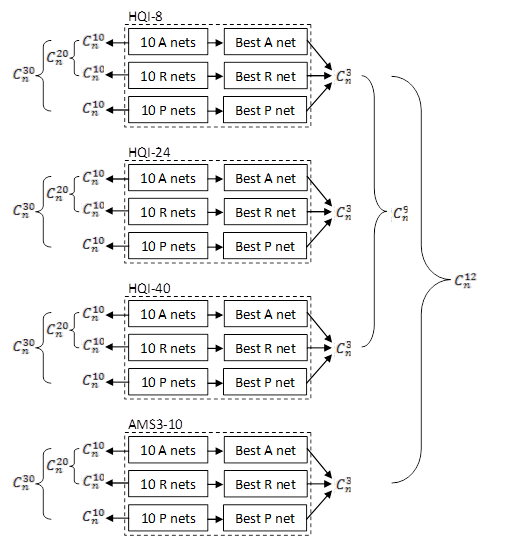

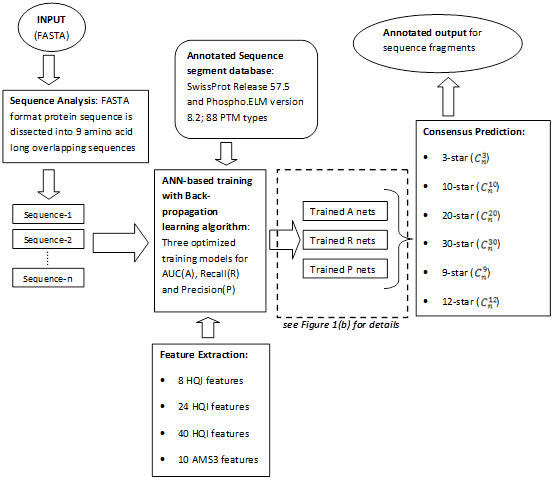
(a) (b)

**Figure 1. (a)** The schematic block diagram of the consensus based prediction server for Post-Translational Modification sites in Protein sequences. **(b)** a detailed description of the consensus algorithm is shown.

acknowledgements

SB is thankful to the Centre for Microprocessor Applications for Training Education and Research (CMATER) and PURSE project of Computer Science and Engineering Department, Jadavpur University, India, for providing necessary infrastructural facilities during the progress of the work. Authors also acknowledge the contributions of many students, researchers and colleagues of CMATER in developing several key modules of the training and prediction routines, now made available in public domain. The authors would like to thank Marcin Kierczak, PhD for his significant help with the automatic processing of the Swiss-Prot database, Prof. Marek Niezgódka and Dr Anna Trykozko for their support of the scientific visit of SB at ICM, University of Warsaw.

*Funding*: This work was supported by EC OxyGreen (KBBE-2007-212281) 6FP project, as well as the Polish Ministry of Education and Science (N301 159735, N518 409238 and others). The calculations were performed in the Interdisciplinary Centre for Mathematical and Computational Modelling (ICM) at Warsaw University.

References

Bairoch, A. and Apweiler, R. (1999) The SWISS-PROT protein sequence data bank and its supplement TrEMBL in 1999, *Nucleic Acids Res.*, **27**, 49-54.

Basu, S., Plewczynski, D. (2010) AMS 3.0: prediction of post-translational modifications. *BMC Bioinformatics* 11: 210

Bezdek, J.C.: Pattern Recognition with Fuzzy Objective Function Algorithms. Plenum, New York (1981)

Blom, N., Gammeltoft, S. and Brunak, S. (1999) Sequence and structure-based prediction of eukaryotic protein phosphorylation sites, *J Mol Biol*, **294**, 1351-1362.

Blom, N., Sicheritz-Ponten, T., Gupta, R., Gammeltoft, S. and Brunak, S. (2004) Prediction of post-translational glycosylation and phosphorylation of proteins from the amino acid sequence, *Proteomics*, **4**, 1633-1649.

Diella, F., Cameron, S., Gemund, C., Linding, R., Via, A., Kuster, B., Sicheritz-Ponten, T., Blom, N. and Gibson, T.J. (2004) Phospho.ELM: a database of experimentally verified phosphorylation sites in eukaryotic proteins, *BMC Bioinformatics*, **5**, 79.

Diella, F., Gould, C.M., Chica, C., Via, A. and Gibson, T.J. (2008) Phospho.ELM: a database of phosphorylation sites--update 2008, *Nucleic Acids Res.*, **36**, D240-244.

Gnad, F., Ren, S., Choudhary, C., Cox, J., and Mann, M. (2010) Predicting post-translational lysine acetylation using support vector machines, *Bioinformatics,* 26(13): 1666–1668.

Gupta, R. and Brunak, S. (2002) Prediction of glycosylation across the human proteome and the correlation to protein function, *Pac Symp Biocomput*, 310-322.

Gupta, R., Jung, E., Gooley, A.A., Williams, K.L., Brunak, S. and Hansen, J. (1999) Scanning the available Dictyostelium discoideum proteome for O-linked GlcNAc glycosylation sites using neural networks, *Glycobiology*, **9**, 1009-1022.

Hjerrild, M., Stensballe, A., Rasmussen, T.E., Kofoed, C.B., Blom, N., Sicheritz-Ponten, T., Larsen, M.R., Brunak, S., Jensen, O.N. and Gammeltoft, S. (2004) Identification of phosphorylation sites in protein kinase A substrates using artificial neural networks and mass spectrometry, *J Proteome Res*, **3**, 426-433.

Julenius, K., Molgaard, A., Gupta, R. and Brunak, S. (2005) Prediction, conservation analysis, and structural characterization of mammalian mucin-type O-glycosylation sites, *Glycobiology*, **15**, 153-164.

Kawashima, S., Kanehisa, M.: AAindex: amino acid index database. *Nucleic Acids Res.* 28, 374 (2000)

Kawashima, S., Ogata, H., Kanehisa, M.: AAindex: amino acid index database. *Nucleic Acids Res.* 27, 368–369 (1999)

Kawashima, S., Pokarowski, P., Pokarowska, M., Kolinski, A., Katayama, T., Kanehisa, M.: AAindex: amino acid index database, progress report 2008. *Nucleic Acids Res.* 36, D202–D205 (2008)

Kim, J.H., Lee, J., Oh, B., Kimm, K. and Koh, I. (2004) Prediction of phosphorylation sites using SVMs, *Bioinformatics (Oxford, England)*, **20**, 3179-3184.

Koenig, M. and Grabe, N. (2004) Highly specific prediction of phosphorylation sites in proteins, *Bioinformatics (Oxford, England)*, **20**, 3620-3627.

Krishnapuram, R., Joshi, A., Yi, L.: A fuzzy relative of the k-medoids algorithm with application to web document and snippet clustering, in *Proceedings of IEEE International Conference Fuzzy Systems* - FUZZ-IEEE 99 pp. 1281–1286 (1999)

Li, S., Li, H., Li, M., Shyr, Y., Xie. L., Li, Y. (2009) Improved prediction of lysine acetylation by support vector machines, *Protein Pept Lett*., 16(8):977-83.

Maulik, U., Saha, I.: Modified differential evolution based fuzzy clustering for pixel classification in remote sensing imagery. *Pattern Recognition* 42(9), 2135–2149 (2009)

Maulik, U., Saha, I.: Automatic fuzzy clustering using modified differential evolution for image classification. *IEEE Transactions on Geoscience and Remote Sensing* 48(9), 3503–3510 (2010)

Maulik, U., Bandyopadhyay, S., Saha, I.: Integrating clustering and supervised learning for categorical data analysis. *IEEE Transactions on Systems, Man and Cybernetics* Part-A 40(4), 664–675 (2010)

Maulik, U., Bandyopadhyay, S.: Fuzzy partitioning using a real-coded variable-length genetic algorithm for pixel classification. *IEEE Transactions on Geoscience and Remote Sensing* 41(5), 1075–1081 (2003)

Monigatti, F., Gasteiger, E., Bairoch, A. and Jung, E. (2002) The Sulfinator: predicting tyrosine sulfation sites in protein sequences, *Bioinformatics (Oxford, England)*, **18**, 769-770.

Nakai, K., Kidera, A., Kanehisa, M.: Cluster analysis of amino acid indices for prediction of protein structure and function. *Protein Engineering* 2, 93–100 (1988)

Plewczynski, D., Tkacz, A., Wyrwicz, L.S. and Rychlewski, L. (2005) AutoMotif server: prediction of single residue post-translational modifications in proteins, *Bioinformatics (Oxford, England)*, **21**, 2525-2527.

Rumelhart, David E., Hinton, Geoffrey E., Williams, Ronald J.: Learning Internal Representations by Error Propagation, *DTIC Document* (1985).

Rumelhart, David E., Hinton, Geoffrey E., Williams, Ronald J.: Learning internal representations by error propagation, *Parallel distributed processing (MIT Press, Cambridge)*, **1**, 318-363 (1986).

Saha, I., Maulik, U., Bandyopadhyay, S., Plewczynski, D. (2011) Fuzzy Clustering of Physicochemical and Biochemical Properties of Amino Acids, *Amino Acids*.

Sigrist, C.J., Cerutti, L., Hulo, N., Gattiker, A., Falquet, L., Pagni, M., Bairoch, A. and Bucher, P. (2002) PROSITE: a documented database using patterns and profiles as motif descriptors, *Briefings in bioinformatics*, **3**, 265-274.

Tomii, K., Kanehisa, M.: Analysis of amino acid indices and mutation matrices for sequence comparison and structure prediction of proteins. *Protein Engineering* 9, 27–36 (1996)

Wan, J., Kang, S., Tang, C., Yan, J., Ren, Y., Liu, J., Gao, X., Banerjee, A., Ellis, L.B. and Li, T. (2008) Meta-prediction of phosphorylation sites with weighted voting and restricted grid search parameter selection, *Nucleic acids research*, **36**, e22.

Wong, Y.H., Lee, T.Y., Liang, H.K., Huang, C.M., Wang, T.Y., Yang, Y.H., Chu, C.H., Huang, H.D., Ko, M.T. and Hwang, J.K. (2007) KinasePhos 2.0: a web server for identifying protein kinase-specific phosphorylation sites based on sequences and coupling patterns, *Nucleic acids research*, **35**, W588-594.

Xu, Y., Wang, X.B., Ding, J., Wu, L.Y., Deng, N.Y. (2010) Lysine acetylation sites prediction using an ensemble of support vector machine classifiers, *J Theor Biol.*, 264(1):130-5.

Xue, Y., Li, A., Wang, L., Feng, H. and Yao, X. (2006) PPSP: prediction of PK-specific phosphorylation site with Bayesian decision theory, *BMC Bioinformatics*, **7**, 163.

Xue, Y., Zhou, F., Zhu, M., Ahmed, K., Chen, G. and Yao, X. (2005) GPS: a comprehensive www server for phosphorylation sites prediction, *Nucleic acids research*, **33**, W184-187.

Yaffe, M.B., Leparc, G.G., Lai, J., Obata, T., Volinia, S. and Cantley, L.C. (2001) A motif-based profile scanning approach for genome-wide prediction of signaling pathways, *Nat Biotechnol*, **19**, 348-353.

**Table 1.** Comparison of AMS-3 and AMS-4 performances all 88 PTM types.

|  | **Single network** | | **AMS-4 Meta-Consensus** |  |  |
| --- | --- | --- | --- | --- | --- |
|  | **AMS-3** | |  | **Gain over AMS-3** | |
| **PTM** | **Average** | **Maximum** | **Maximum** | **Average** | **Maximum** |
| **Phosphothreonine_CDC2** | 0.685068 | 0.698365 | 0.910212 | 32.864475 | 30.33471 |
| **GRK_group** | 0.614195 | 0.693856 | 0.776483 | 26.422879 | 11.908379 |
| **CK1_group** | 0.4375 | 0.4375 | 0.541667 | 23.8096 | 23.8096 |
| **AMPK_group** | 0.769388 | 0.77551 | 0.94898 | 23.342189 | 22.368506 |
| **Abl** | 0.689333 | 0.693333 | 0.833333 | 20.889759 | 20.192317 |
| **Lyn** | 0.676389 | 0.680556 | 0.805556 | 19.096555 | 18.367335 |
| **Phosphoserine** | 0.734679 | 0.769004 | 0.865732 | 17.838131 | 12.578348 |
| **Tyrosine** | 0.81172 | 0.827492 | 0.954545 | 17.595353 | 15.353985 |
| **PLK1** | 0.729353 | 0.743781 | 0.854892 | 17.212379 | 14.938671 |
| **MAPK14** | 0.738125 | 0.74375 | 0.8625 | 16.850127 | 15.966387 |
| **GSK-3_group** | 0.747253 | 0.747253 | 0.870879 | 16.544062 | 16.544062 |
| **PDK-1** | 0.7375 | 0.8125 | 0.854167 | 15.819254 | 5.1282462 |
| **MAPKAPK2** | 0.647619 | 0.649471 | 0.743386 | 14.787553 | 14.46023 |
| **ATM** | 0.83347 | 0.842033 | 0.950549 | 14.047176 | 12.887381 |
| **Syk** | 0.685417 | 0.6875 | 0.770833 | 12.461903 | 12.121164 |
| **IGF1R** | 0.475 | 0.475 | 0.530556 | 11.696 | 11.696 |
| **Glutamate** | 0.895833 | 0.895833 | 0.994792 | 11.04659 | 11.04659 |
| **Glutamate_methyl** | 0.895833 | 0.895833 | 0.994792 | 11.04659 | 11.04659 |
| **Blocked_amino_end_Met** | 0.905039 | 0.905039 | 1 | 10.492476 | 10.492476 |
| **MAPK8** | 0.858201 | 0.863757 | 0.944444 | 10.049278 | 9.3414004 |
| **Asparagine** | 0.904029 | 0.905678 | 0.994505 | 10.008086 | 9.8077904 |
| **PKC_alpha** | 0.753903 | 0.768086 | 0.820959 | 8.894513 | 6.8837344 |
| **CaM-KIIalpha** | 0.804825 | 0.804825 | 0.876096 | 8.8554655 | 8.8554655 |
| **InsR** | 0.631944 | 0.638889 | 0.6875 | 8.7912853 | 7.6086769 |
| **allempty** | 0.713086 | 0.722945 | 0.772032 | 8.2663241 | 6.7898664 |
| **PKA_alpha** | 0.89011 | 0.89011 | 0.961538 | 8.0246262 | 8.0246262 |
| **Glycine** | 0.923046 | 0.923046 | 0.997076 | 8.0201853 | 8.0201853 |
| **Phosphothreonine_autocatalysis** | 0.779718 | 0.780316 | 0.837898 | 7.461672 | 7.3793181 |
| **Src** | 0.738434 | 0.74128 | 0.791306 | 7.160017 | 6.748597 |
| **Phosphoserine_CK2** | 0.83826 | 0.846481 | 0.893079 | 6.5396178 | 5.504908 |
| **Lck** | 0.82862 | 0.831089 | 0.882716 | 6.5284449 | 6.2119701 |
| **Phosphoserine_autocatalysis** | 0.777933 | 0.782634 | 0.826146 | 6.1975774 | 5.5596869 |
| **PKB_group** | 0.882353 | 0.882353 | 0.933824 | 5.8333796 | 5.8333796 |
| **methylated_arginine** | 0.914561 | 0.934175 | 0.967662 | 5.8061737 | 3.5846603 |
| **acetylthreonine** | 0.93125 | 0.952206 | 0.985294 | 5.8033826 | 3.4748783 |
| **Lysine_methyl** | 0.904635 | 0.904635 | 0.957127 | 5.8025613 | 5.8025613 |
| **Leucine** | 0.917082 | 0.917082 | 0.970064 | 5.7772369 | 5.7772369 |
| **MAPK3** | 0.903863 | 0.903863 | 0.955663 | 5.730957 | 5.730957 |
| **Phospho_CDC2** | 0.878211 | 0.881292 | 0.926418 | 5.4892275 | 5.1204368 |
| **Fyn** | 0.649079 | 0.677632 | 0.684211 | 5.4125923 | 0.970881 |
| **Phosphoserine_PKA** | 0.901028 | 0.90653 | 0.946926 | 5.0939593 | 4.4561129 |
| **acetyllysine** | 0.93525 | 0.9355 | 0.98275 | 5.0788559 | 5.050775 |
| **Glycine_amide** | 0.9125 | 0.933333 | 0.958333 | 5.0227945 | 2.6785724 |
| **Phosphoserine_PKC** | 0.899708 | 0.901542 | 0.944809 | 5.0128486 | 4.7992218 |
| **Phosphotyrosine_autocatalysis** | 0.878906 | 0.887277 | 0.921875 | 4.8889187 | 3.899346 |
| **dihydroxyphenylalanine** | 0.859375 | 0.859375 | 0.898438 | 4.5455127 | 4.5455127 |
| **Lysine_trimethyl** | 0.809692 | 0.859487 | 0.846154 | 4.5031938 | -1.551274 |
| **EGFR** | 0.711875 | 0.725 | 0.74375 | 4.4776119 | 2.5862069 |
| **4-carboxyglutamate** | 0.902848 | 0.915701 | 0.941563 | 4.2880972 | 2.8242843 |
| **CDK2** | 0.915536 | 0.916384 | 0.953955 | 4.1963396 | 4.0999188 |
| **3-4-dihydroxyproline** | 0.49902 | 0.519608 | 0.519608 | 4.1256863 | 0 |
| **Phospho_PKA** | 0.915226 | 0.926318 | 0.951779 | 3.9938769 | 2.7486241 |
| **Phospho_PKC** | 0.881779 | 0.890336 | 0.915196 | 3.7897251 | 2.7922043 |
| **Phospho_auto** | 0.90412 | 0.911261 | 0.937841 | 3.729704 | 2.9168372 |
| **acetylmethionine** | 0.96267 | 0.962928 | 0.994832 | 3.3409164 | 3.313228 |
| **CDK_group** | 0.920976 | 0.920976 | 0.95092 | 3.2513334 | 3.2513334 |
| **Phosphoserine_CaMK2** | 0.839286 | 0.839286 | 0.866071 | 3.1914032 | 3.1914032 |
| **CK2_alpha** | 0.85 | 0.853368 | 0.875959 | 3.054 | 2.6472753 |
| **CaM-KII_group** | 0.838068 | 0.852273 | 0.863636 | 3.0508264 | 1.3332582 |
| **5-hydroxylysine** | 0.905274 | 0.905274 | 0.931645 | 2.9130407 | 2.9130407 |
| **PKC_group** | 0.832146 | 0.838365 | 0.856301 | 2.9027358 | 2.1394023 |
| **MAPK1** | 0.920052 | 0.920779 | 0.945974 | 2.8174494 | 2.73627 |
| **MAPK_group** | 0.892817 | 0.892817 | 0.917508 | 2.7655163 | 2.7655163 |
| **Valine** | 0.970238 | 0.970238 | 0.997024 | 2.7607659 | 2.7607659 |
| **CDK1** | 0.942908 | 0.942908 | 0.968085 | 2.6701439 | 2.6701439 |
| **Methionine** | 0.969853 | 0.977941 | 0.992647 | 2.3502531 | 1.5037717 |
| **4-hydroxyproline** | 0.889265 | 0.89575 | 0.910114 | 2.3445205 | 1.6035724 |
| **Blocked_amino_end** | 0.833397 | 0.836538 | 0.852564 | 2.2998643 | 1.9157528 |
| **Sulfotyrosine** | 0.919695 | 0.926131 | 0.940837 | 2.2988056 | 1.5878963 |
| **IKK_group** | 0.533036 | 0.544643 | 0.544643 | 2.1775265 | 0 |
| **Pyruvic** | 0.965216 | 0.967723 | 0.983776 | 1.9228857 | 1.6588425 |
| **CK2_group** | 0.866406 | 0.867188 | 0.882813 | 1.8936849 | 1.8018008 |
| **Phenylalanine** | 0.962111 | 0.968141 | 0.980239 | 1.8841901 | 1.2496114 |
| **GSK-3beta** | 0.715225 | 0.715225 | 0.72782 | 1.7609843 | 1.7609843 |
| **Phosphoserine_CDC2** | 0.881649 | 0.881649 | 0.897163 | 1.7596572 | 1.7596572 |
| **Cysteine** | 0.972738 | 0.972738 | 0.989538 | 1.7270838 | 1.7270838 |
| **acetylserine** | 0.973851 | 0.989224 | 0.989224 | 1.5785782 | 0 |
| **Phosphotyrosine** | 0.885021 | 0.889636 | 0.896274 | 1.2714953 | 0.7461479 |
| **dimethylarginine** | 0.958458 | 0.959281 | 0.969869 | 1.1905582 | 1.1037433 |
| **aspartylphosphate** | 0.986897 | 0.98761 | 0.998217 | 1.1470295 | 1.0740069 |
| **Hydroxyproline** | 0.818038 | 0.821118 | 0.825932 | 0.9649918 | 0.5862738 |
| **PKA_group** | 0.879249 | 0.881895 | 0.886621 | 0.8384428 | 0.5358915 |
| **Phosphothreonine** | 0.878275 | 0.880452 | 0.885382 | 0.8091999 | 0.5599397 |
| **Pyrrolidone** | 0.9865 | 0.989286 | 0.989286 | 0.2824126 | 0 |
| **acetylglycine** | 0.767857 | 0.767857 | 0.767857 | 0 | 0 |
| **Allysine** | 0.924425 | 0.924425 | 0.924425 | 0 | 0 |
| **Cysteine_amide** | 0.946795 | 0.946795 | 0.946795 | 0 | 0 |
| **Cysteine_methyl** | 0.989177 | 0.989177 | 0.989177 | 0 | 0 |

**Table 2.**Benchmark results of the consensus prediction for selected four major types of phosphorylation.

|  | **CDK_group** | **CK2_group** | **PKA_group** | **PKC_group** |
| --- | --- | --- | --- | --- |
| **GPS** | *0.87* | *0.81* | *0.84* | *0.75* |
| **KinasePhos** | *0.87* | *0.75* | *0.82* | *0.74* |
| **NetPhosK** | *0.77* | ***0.93*** | *0.87* | *0.75* |
| **PPSP** | *0.87* | *0.87* | *0.88* | *0.79* |
| **PredPhospho** | *0.86* | *0.77* | *0.85* | *0.71* |
| **Scansite** | *0.75* | *0.77* | *0.76* | *0.63* |
| **Meta-predictor** | *0.89* | ***0.93*** | ***0.89*** | *0.82* |
| **AMS 3.0** | 0.92 | 0.87 | 0.88 | 0.84 |
| **AMS 4.0** | **0.95** | 0.88 | **0.89** | **0.86** |
